# Ossifying fasciitis of the hand: case report and review of diagnostic pitfalls

**DOI:** 10.1080/23320885.2026.2695498

**Published:** 2026-06-27

**Authors:** Azadeh Rakhshan, Babak Toloue Ghamari, Negin Rajabi, Sara Sadeghzadeh, Hanieh Zham

**Affiliations:** a Department of Pathology, Shohadae Tajrish Hospital, School of Medicine, Shahid Beheshti University of Medical Sciences, Tehran, Iran; b Department of Orthopedic Surgery, Clinical Research Development Unit of Shohadae Tajrish Hospital, Shahid Beheshti University of Medical Sciences, Tehran, Iran; c Student Research Committee, School of Medicine, Shahid Beheshti University of Medical Sciences, Tehran, Iran

**Keywords:** Ossifying fasciitis, palmar mass, nodular fasciitis, hand tumor, case report

## Abstract

Ossifying fasciitis represents an uncommon benign lesion within the spectrum of reactive fibroblastic proliferations. It is defined by the coexistence of spindle-cell proliferation and heterotopic bone formation, a combination that can easily simulate malignant soft-tissue tumors. Because of its rapid growth and radiologic density, it is frequently mistaken for sarcomatous processes. Although most cases occur in the trunk and proximal extremities, presentation within the hand is exceptionally rare. We describe a 34-year-old male who developed a painless nodule on the palmar aspect of the right hand growing over two months. Imaging revealed a well-circumscribed lesion with heterogeneous signal intensity adjacent to the flexor tendon. The excised mass was composed of fibroblasts and myofibroblasts arranged in fascicles within a fibromyxoid stroma, showing osteoid formation rimmed by osteoblasts and scattered multinucleated giant cells. No cytologic atypia or atypical mitoses were present. Immunohistochemistry demonstrated diffuse smooth-muscle-actin (SMA) positivity and absence of H-caldesmon and ALK expression, confirming a diagnosis of ossifying fasciitis. This case underscores the diagnostic pitfalls of ossifying fasciitis, particularly when arising in unusual anatomic locations. Awareness of its characteristic histologic features and correlation with clinical and imaging findings is crucial to avoid misinterpretation as a malignant neoplasm and to prevent unnecessary radical surgery. Reporting such cases expands understanding of their variable presentations and contributes to refining clinical decision-making.

## Introduction

Ossifying fasciitis is a rare, benign soft tissue lesion, considered a histologic variant of nodular fasciitis [1]. It is characterized by reactive fibroblastic proliferation and the presence of heterotopic bone formation [2]. The cause remains unclear, but it is likely triggered by trauma or infection [3]. Although primarily seen in young adults, several studies have reported it in pediatric patients [1]. The lesion is typically located within the fascia or skeletal muscle, most commonly affecting the head and neck, upper extremities, back, and chest regions [2]. Ossifying fasciitis is notable for its rapid growth, often developing into a heterotopic ossifying mass within a few weeks. As a result, the lesion can be easily misdiagnosed as a malignancy [4]. Hereby, we present a case of a 34-year-old man with the uncommon presentation of ossifying fasciitis on the palmar surface of the hand, highlighting the clinical, radiological, and histopathological features that led to this diagnosis and discussing the importance of distinguishing it from malignant pathologies to prevent unnecessarily aggressive wide resections.

## Patient

**Figure 1.**
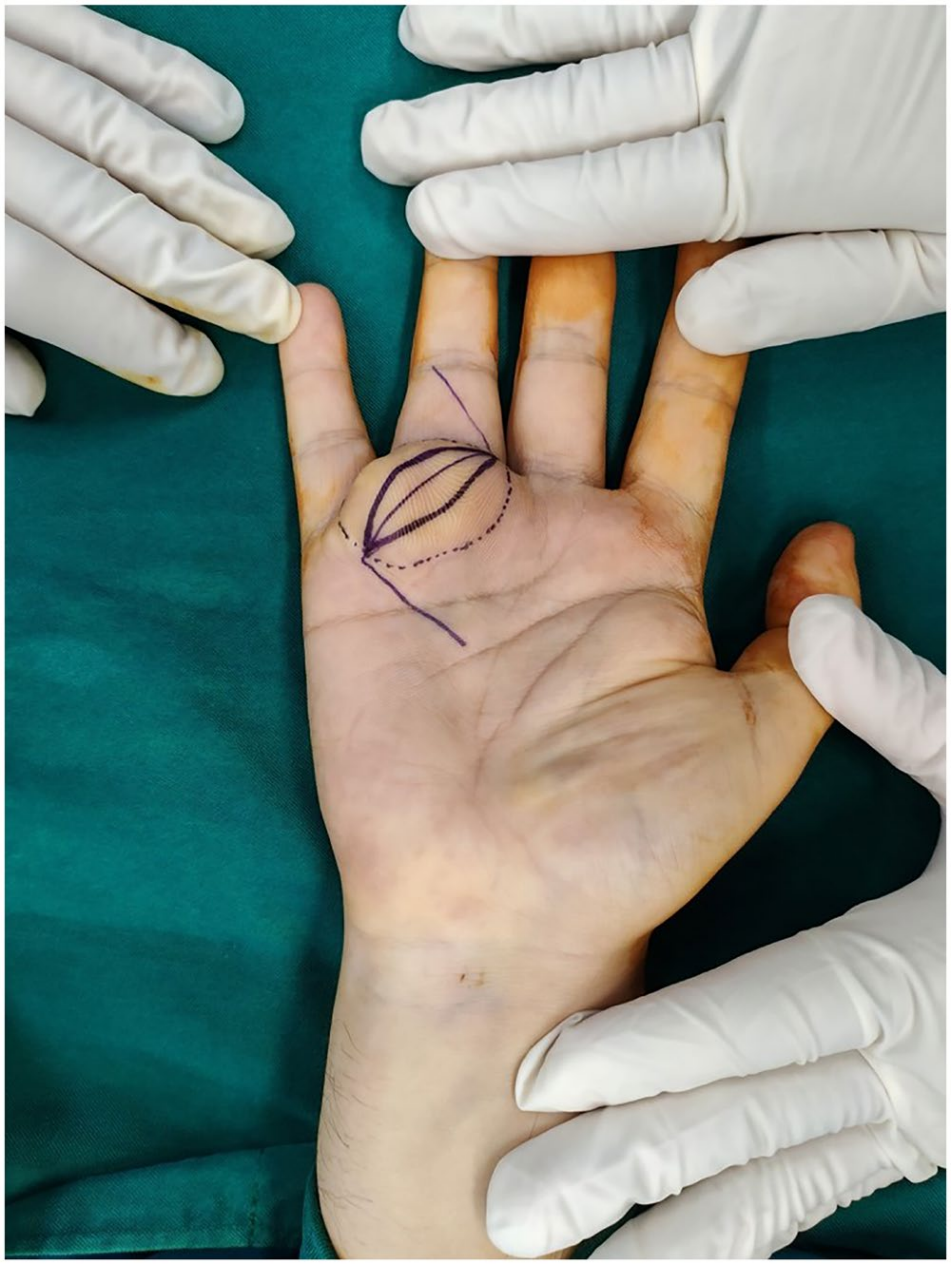
The bulging mass on the palmar aspect of the right hand.

A 34-year-old right-handed male presented to our hospital with a two-month history of a progressively enlarging mass located on the palmar aspect of the right hand (Figure 1). He denied any history of trauma, local injections, or prior surgeries involving the hand; however, it is noteworthy that his occupation involved manual labor and frequent exposure to construction site environments. The patient did not report any paresthesia; however, while the mass remained asymptomatic at rest, he experienced mild discomfort during hand movements. Approximately 40 days before presentation at our center, the patient had undergone a core needle biopsy at another medical facility. The histopathologic diagnosis at that time was nodular tenosynovitis (giant cell tumor of the tendon sheath). However, as the lesion was small and asymptomatic, the patient did not seek any further medical evaluation or treatment at that time. Over the following weeks, the lesion demonstrated noticeable and rapid growth. Concerned by the increase in size, the patient sought re-evaluation and presented to our hospital for further assessment and management.

**Figure 2.**
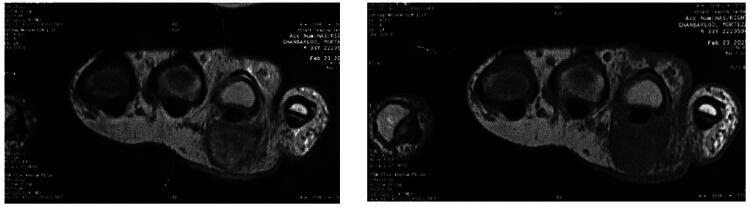
MRI scans of the right hand in axial planes showing a well-defined mass with low signal intensity on T1-weighted image (right side) and high signal intensity on T2-weighted image (left side), located on the palmar aspect of the fourth finger and surrounding the flexor tendon.

On clinical examination, a prominent mass was palpated on the volar surface of the right hand, situated in close proximity to the fourth metacarpophalangeal joint. It measured approximately 4.0 × 1.5 cm, had an elastic-to-firm consistency, and was mobile on palpation. Range of motion was mostly preserved, with slight limitation at terminal flexion of the fourth and fifth metacarpophalangeal joints. There was no overlying skin change, warmth, or erythema. Neurological and vascular examination of the hand was unremarkable. The MRI of the patient shows a mildly heterogeneous low T1, high T2 mass on the palmar aspect of the fourth finger, extending around the adjacent flexor tendon (Figure 2).

## Results

**Figure 3.**
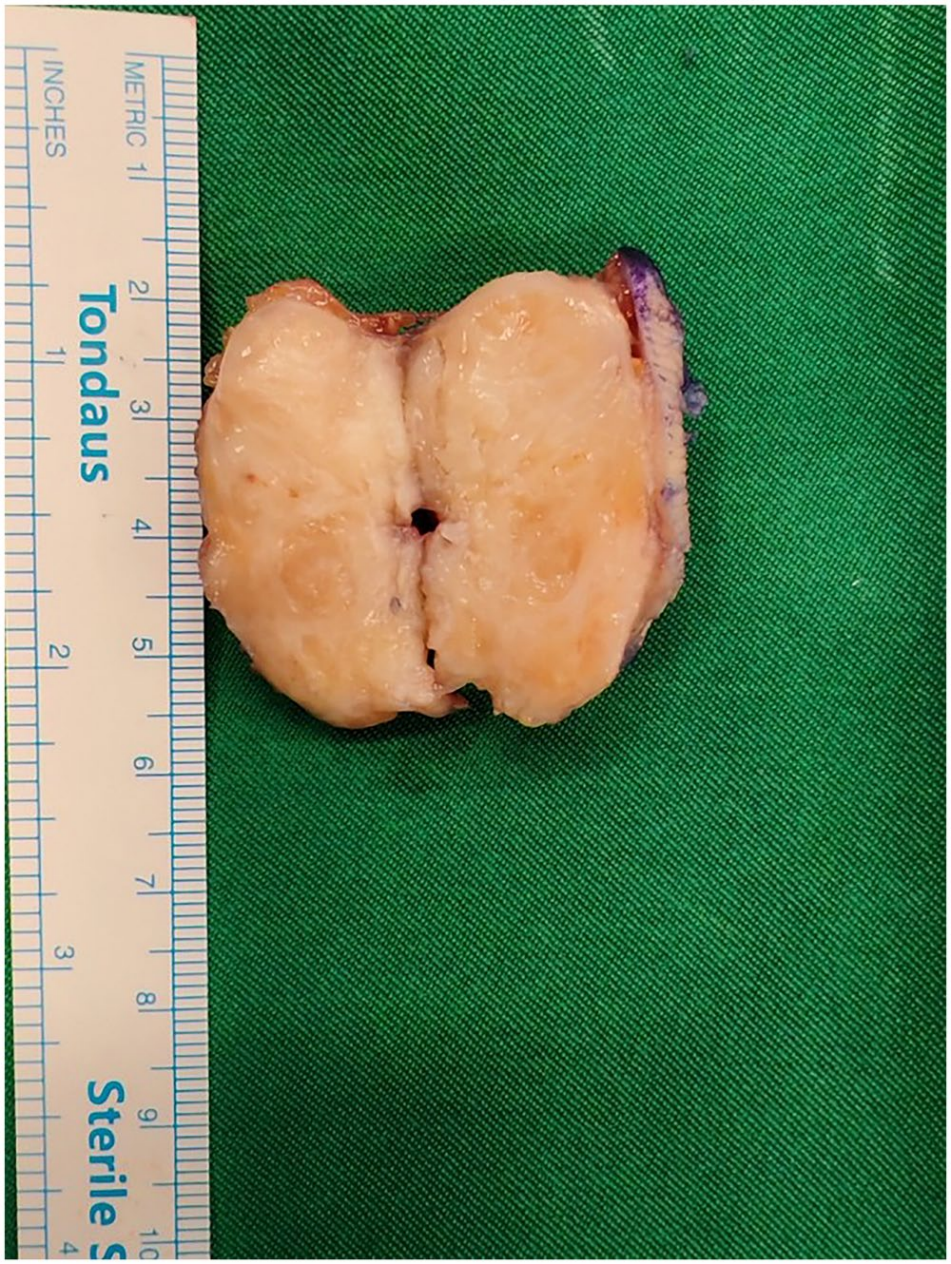
Gross specimen of the resected palmar mass measuring approximately 3.5 × 2 × 2 cm, with a smooth external surface and homogeneously tan cut section.

The patient subsequently underwent surgical excision of the lesion under regional anesthesia. In the operating room, the patient was placed supine. We used a pneumatic tourniquet on the middle arm. Under regional axillary and musculocutaneous anesthesia, the incision was initiated in a Z-plasty manner at the volar level. The biopsy tract was excised elliptically along the incision. Intraoperatively, the digital nerve and blood vessels located in the deeper part of the mass were carefully explored and separated from the mass. The encapsulated mass was observed to be protruding from the flexor tendon sheath and the A1 pulley. After mobilizing the neurovascular bundle, the tumor was carefully isolated and excised with marginal resection (Figure 3).

**Figure 4.**
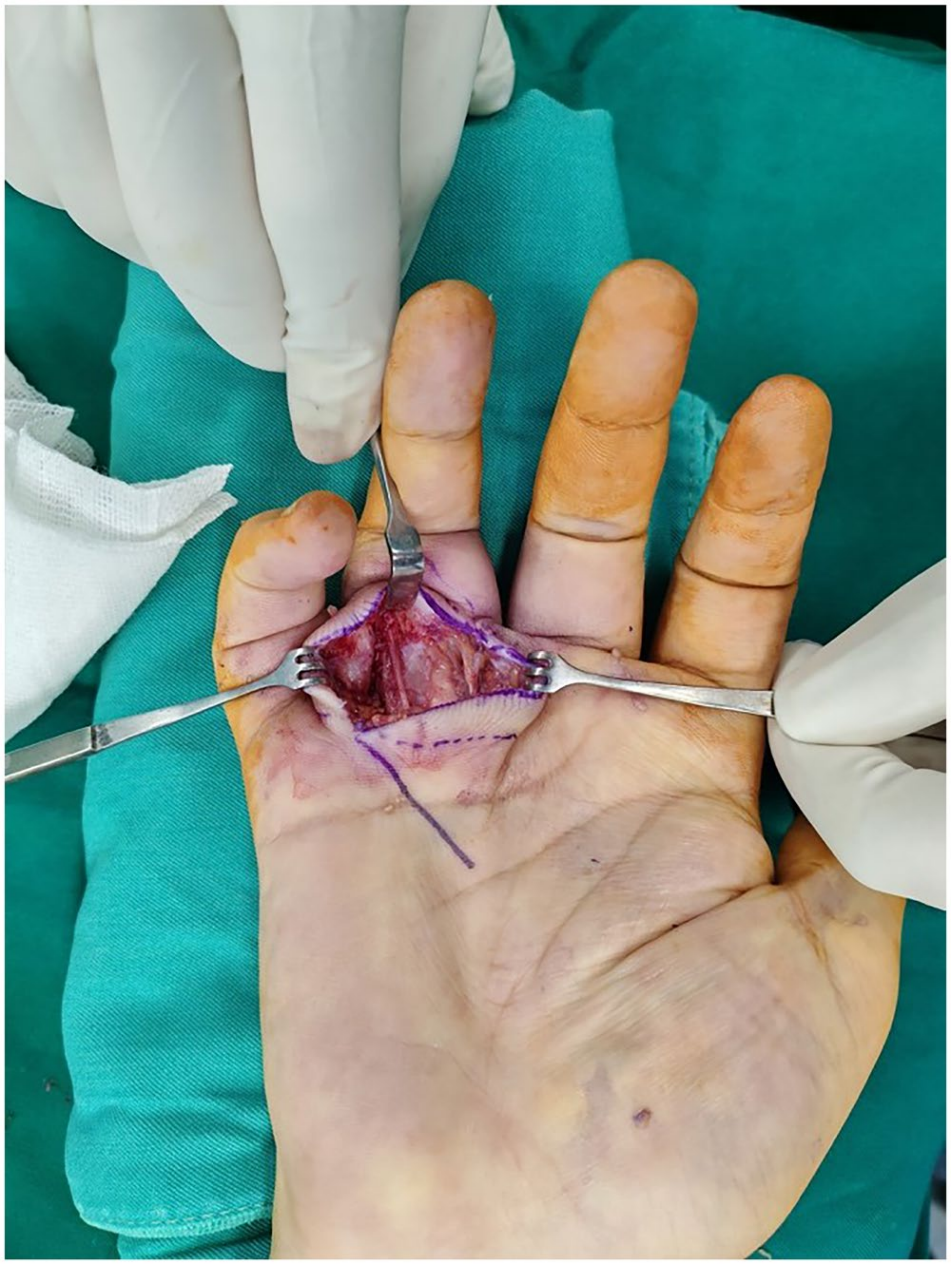
Intraoperative image demonstrating the exposed palmar mass during surgical excision on the right hand.

Gross examination revealed an elliptical skin segment with an attached subcutaneous ovoid, tan, elastic-to-firm mass measuring 3.5 × 2 × 2 cm. The cut surface was homogeneously tan (Figure 4).

**Figure 5.**
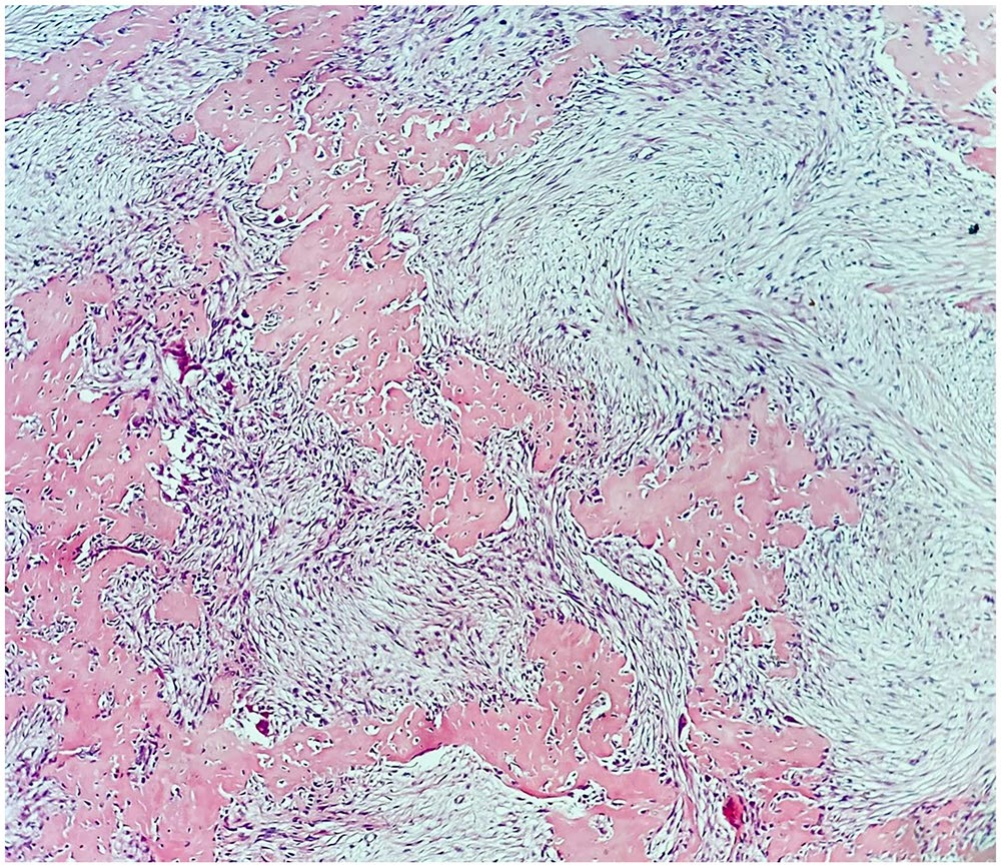
Microscopic image shows spindle cell proliferation with variable cellularity arranged in loose fascicular and storiform pattern. Stroma is myxoid and fibrocollagenous. Extensive osteoid formation and scattered osteoclast type giant cells are noted (Hematoxylin and Eosin stain, x200 magnification).

Microscopic evaluation showed a non-encapsulated, well-circumscribed neoplasm located in the deep dermis and subcutaneous tissue. The lesion was composed of plump fibroblasts and myofibroblast-like spindle cells with minimal cytologic atypia and low mitotic activity embedded in a fibrocollagenous and mildly myxoid stroma. A few scattered osteoclasts were present, with no evidence of necrosis or atypical mitotic figures. Significant osteoid formation was noted, characterized by an active rim of osteoblasts and localized areas of mineralization (Figure 5).

Immunohistochemistry (IHC) showed the tumor cells were positive for smooth muscle actin (SMA) in a diffuse pattern, positive for CD68 in a patchy pattern, and negative for both H-caldesmon and ALK

At the six-month follow-up, the patient remained asymptomatic and had successfully resumed his occupation as a construction worker. Clinical examination demonstrated a full range of motion of the right hand without pain on active or passive movements. Sensory and motor neurological examinations were normal, with no evidence of tendon dysfunction. There was no clinical evidence of local recurrence.

## Discussion

Ossifying fasciitis is a rare variant of nodular fasciitis characterized by the presence of metaplastic bone formation within the lesion [1]. First described as a distinct entity by Kwitkken and Branche in 1969, it often clinically and histologically mimics soft tissue malignant tumors, leading to potential misdiagnosis and overtreatment [5]. Despite the worrisome features, ossifying fasciitis retains the self-limiting and reactive nature of conventional nodular fasciitis [1].

A review of recent PubMed-reported cases (Table 1) revealed that approximately half of the cases documented a history of trauma [2,4,6], whereas the other half did not report a definite trauma [5,7,8] This supports the hypothesis that trauma may act as a triggering factor, but is not essential for lesion development and pathogenesis [1]. This was consistent with our patient, who reported no significant trauma to the affected hand.

**Table 1.** Summary of the characteristics, clinical finding, and outcome measures of the previously reported cases.

Study	Patient age/sex	Location	Duration	History of trauma	Pain	Mobility
Kwittken & Branche [5]	24 males	Right thigh	2 months	Reported possible minor trauma caused by cartons hitting thigh	No pain	Slightly mobile
Kim et al. [4]	23 males	Left hand	2 months	Falling and minor trauma 2 months prior	Painful with movement	Mobile
Dogan et al. [6]	57 males	Left femoral region	3 months	Prior angiography catheterization (trauma)	Painful with movement	Not specified
Kowalczyk et al. [2]	30 females	Left knee	1.5 months	Reported trauma 6 weeks prior	painful	Not specified
Hammoutene et al. [7]	32, sex not mentioned	Chin	2 years	None reported	No pain	Mobile
Bormann et al. (8)	23 females	Left proximal thigh (vulvar crease)	2 months	None reported	Painful with movement	Not specified

Pain presentation is variable among cases; some cases are entirely painless [5,7], while others demonstrate mild tenderness or pain upon movement [2,4,6,8]. In our patient, the lesion was not painful. Importantly, neurological symptoms are typically absent, further distinguishing it from invasive malignant processes [1].

The duration of the lesion is usually short, with most cases having lesions present for about two months, highlighting the rapid growth characteristic of these tumors [2,4–6,8]. Clinically, lesions tend to be mobile or slightly mobile, which serves as a useful distinguishing feature from more infiltrative malignant soft tissue tumors that are typically firm and fixed to underlying structures, reinforcing their benign reactive behavior [4,5,7].

## Diagnostic challenges

Given the overlapping clinical and radiologic features of ossifying fasciitis with other soft tissue lesions, a careful comparison with entities such as desmoid fibromatosis, myositis ossificans, extraskeletal osteosarcoma, and nodular fasciitis is essential for accurate diagnosis.

Desmoid fibromatosis (DF) is a rare, locally aggressive connective tissue tumor arising from musculoaponeurotic structures. Histologically, DF is composed of long seeping fascicles of uniform spindle cells with mild to moderate cellularity within a collagenous stroma, exhibiting minimal atypia and nuclear β-catenin positivity, and on MRI typically demonstrates T2-hypointense fibrotic areas [9]. Unlike DF, ossifying fasciitis shows reactive myofibroblastic proliferation with active osteoid formation. Desmoid-type fibromatosis also has infiltrative margins and a higher risk of local recurrence, distinguishing it from the self-limited and reactive nature of ossifying fasciitis [10,11].

Myositis ossificans is a benign, reactive lesion characterized by heterotopic bone formation, often following trauma, predominantly affecting young active males. It exhibits a zonal histologic pattern with a central fibroblastic core, intermediate woven bone, and peripheral mature lamellar bone [12]. MO typically exhibits peripheral maturation of bone evident on imaging and histology, which contrasts with the more irregular and centrally distributed osteoid formation seen in ossifying fasciitis [12,13].

Extraskeletal osteosarcoma (ESOS) is a rare malignant soft tissue neoplasm with variable histology, including osteoblastic, fibroblastic, chondroblastic, and small cell patterns, marked cellularity, and frequent mitoses, including atypical mitotic figures and commonly necrosis; features that are absent in ossifying fasciitis, while radiographically appearing as a soft tissue mass with heterogeneous calcification or ossification [14]. Unlike ESOS, ossifying fasciitis is self-limited, lacks significant atypia or mitotic activity, and does not infiltrate or metastasize.

Conventional nodular fasciitis shares rapid growth and benign reactive myofibroblastic proliferation but lacks heterotopic bone formation, which represents a defining histopathologic feature of ossifying fasciitis [1,15].

Our case was initially misdiagnosed as a giant cell tumor of the tendon sheath, emphasizing the diagnostic difficulties presented by ossifying fasciitis. Limited biopsy material may not adequately capture the architectural features necessary to distinguish ossifying fasciitis from giant cell tumor of the tendon sheath. Both entities can exhibit a benign proliferation with scattered osteoclast-like giant cells and often arise in close proximity to tendon structures of the hand. Only on excisional biopsy did the characteristic features of ossifying fasciitis, namely, a myofibroblastic proliferation with active osteoid formation rimmed by osteoblasts, become evident. Furthermore, lack of mononuclear histiocyte-like cells, inflammatory cells and hemosiderin, as well as taking growth rate into account (rapid growth of ossifying fasciitis in contrast to slow growth of Giant cell tumor of tendon sheath) allowing for a definitive diagnosis. Accurate differentiation of this tumor from malignant soft tissue neoplasms is critical, as its clinical and pathological features may closely resemble those of sarcomas, but its management and prognosis differ significantly. Therefore, increased awareness and careful clinicopathologic correlation are essential to avoid overtreatment and ensure appropriate patient care.

## Conclusion

Ossifying fasciitis, although rare, should be considered in the differential diagnosis of rapidly enlarging, mobile soft tissue lesions of the hand. Early recognition, supported by clinicopathologic correlation and characteristic histopathologic findings, is critical to prevent misdiagnosis and overtreatment. Reporting such cases enhances awareness among physicians and pathologists, aiding in the establishment of a more accurate diagnostic approach.

## Data Availability

All data analyzed during this study are included in the published article.
